# Complex endoleak treatment after failed endovascular aortic repair

**DOI:** 10.1186/s42155-023-00381-y

**Published:** 2023-07-05

**Authors:** Jan Raupach, Jan Masek, Sindharta Venugopal, Ondrej Renc, Michal Lesko, Maly Radovan

**Affiliations:** 1 Department of Radiology, University Hospital Hradec Kralove, Sokolska 581, Hradec Kralove, 50005 Czech Republic; 2 Faculty of Medicine in Hradec Kralove, Radiology, Charles University, Hradec Kralove, Czech Republic; 3 Department of Surgery, University Hospital Hradec Kralove, Hradec Kralove, Czech Republic; 4 Faculty of Medicine in Hradec Kralove, Surgery, Charles University, Hradec Kralove, Czech Republic; 5 The 1st Department of Internal Medicine – Cardioangiology, University Hospital Hradec Kralove, Hradec Kralove, Czech Republic; 6 Faculty of Medicine in Hradec Kralove, Internal Medicine, Charles University, Hradec Kralove, Czech Republic

## Abstract

**Background:**

Endovascular aneurysm repair (EVAR) has created new possibilities for patients with abdominal aortic aneurysms (AAAs), and in recent years it has become tremendously popular. Use of EVAR in selected groups of patients allows mortality and morbidity to be reduced in comparison to open repair. However, complications such as endoleaks (ELs) can be of great concern and warrant urgent therapy to prevent sac rupture.

**Case presentation:**

The case report presents urgent endovascular treatment of a high-risk type IA EL in a polymorbid 68-year-old patient 7 years after primary EVAR. The principle of treatment was parallel implantation of the proximal SG extension with the renal SG into the right renal artery (chimney technique). The subsequent type II collateral EL was treated by direct transabdominal AAA sac puncture and thrombin embolization.

**Conclusion:**

EL can be a cause for urgent intervention, but specific anatomic features often require specialized SG types which are not readily available. The chimney technique allows the use of immediately available stent grafts to address endoleak in the setting of impending abdominal aneurysm rupture.

## Introduction

In order to reduce the invasiveness and lethality of treatment of unruptured abdominal aneurysms (AAA), endovascular (EV) treatment (Endovascular Aortic Repair—EVAR) was introduced into clinical practice in the 1990s [1]. The principle of this minimally invasive technique is to exclude the aneurysm using an endoluminal prosthesis, the stent graft (SG), which is inserted transarterially via the femoral artery.

Although EVAR is less risky, it is burdened by a higher incidence of late complications [2]. The most common complications specific to EVAR include SG migration and secondary blood leakage into the AAA pouch—endoleak (EL). Years of experience with EVAR have led to the development of new generations of SG and a significant reduction in the incidence of complications [3]. Thus, EL remains as the most common complication of EVAR today, often requiring further endovascular or surgical reintervention [4, 5].

In relation to the danger of AAA rupture, ELs are divided into high-risk types (IA, IB, III) requiring urgent treatment, and low-risk types (collateral, type II) which, if not leading to significant AAA sac progression, can be treated conservatively [6].

## Case presentation

A polymorbid, 68-year-old man with a solitary left kidney and an ejection fraction of 25% underwent percutaneous insertion of a bifurcated SG into the subrenal AAA (sac width of 58 mm). Considering the short subrenal neck (12 mm in length, 24 mm in diameter), a type of SG with a suprarenal fixation of 28 mm in diameter (Endurant, Medtronic Inc., USA) was selected. The inferior mesenteric artery (IMA) and the L4 lumbar artery were embolized with coils to prevent the development of type II EL. The procedure was uneventful, the left solitary renal artery (RA) remained patent and no EL or progression of AAA sac size was evident on follow-up CTA (1 and 3 years after treatment). CTA examinations at 1 and 3 years after EVAR were negative without endoleak and even showed a reduction of the AAA sac from 58 to 55 mm. Subsequently, the patient was monitored annually by ultrasound, which showed stable AAA size until the 6th year after treatment.

**Fig. 1:**
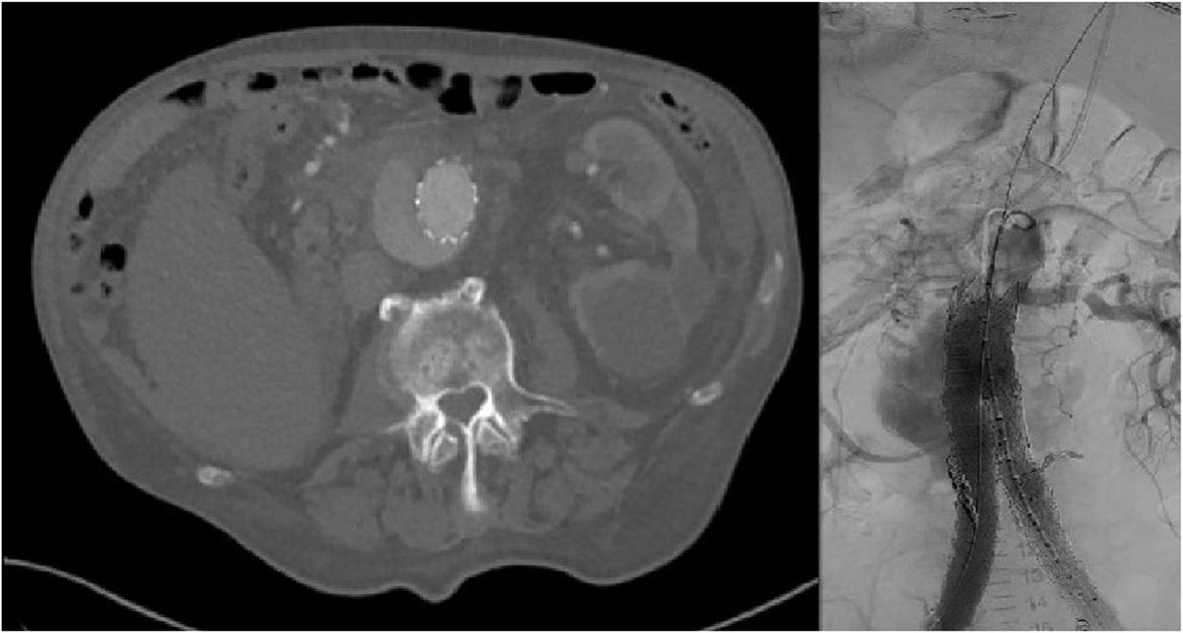
Type IA endoleak presenting 7 years after primary therapy (CTA (a), DSA (b))

**Fig. 2:**
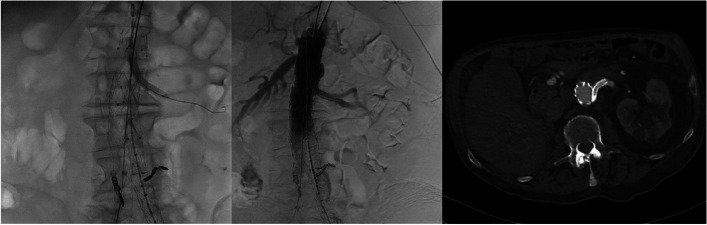
Chimney technique. Dilatation of the renal stent graft after deployment of the proximal aortic extension (DSA) (a). Elimination of type IA endoleak with a tubular extension and stent graft inserted into the solitary renal artery (DSA (b), CTA (c))

At a 7-year follow-up, the patient presented with sudden onset of severe abdominal pain and shortness of breath. Ultrasound examination showed enlargement of the AAA sac and blood leakage at the proximal SG end. Urgent CTA demonstrated a type IA EL and distal migration of the SG along with marked progression of the AAA sac diameter to 91 mm (Fig. 1). Due to high risk of sac rupture, urgent endovascular therapy under analgosedation was indicated. Using a percutaneous approach from the right femoral artery, a 36 × 70 mm tubular SG extension (Endurant, Medtronic Inc., USA) was introduced via a 22F sheath. The distance between the superior mesenteric artery (SMA) and the RA was 30 mm. Simultaneously, a 70 cm long 7F sheath (Cook, Holland) was percutaneously introduced via the left brachial artery. A Rosen guidewire (Cook, The Netherlands) was used to ensure a stable position in the RA. In the lateral projection, the proximal aortic SG extension was first released, starting its covered portion just below the SMA origin and overlapping the left RA origin. Subsequently, in the anteroposterior projection, a 7 × 58 mm balloon-expandable SG (Advanta V12, Atrium Medical Corp., USA) was inserted into the RA parallel with the proximal end at the upper edge of the covered portion of the aortic SG extension. After simultaneous dilatation of the aortic extension and the renal SG, angiography was performed to demonstrate free patency of the RA and elimination of the EL at the proximal abdominal SG portion (Fig. 2).

**Fig. 3:**
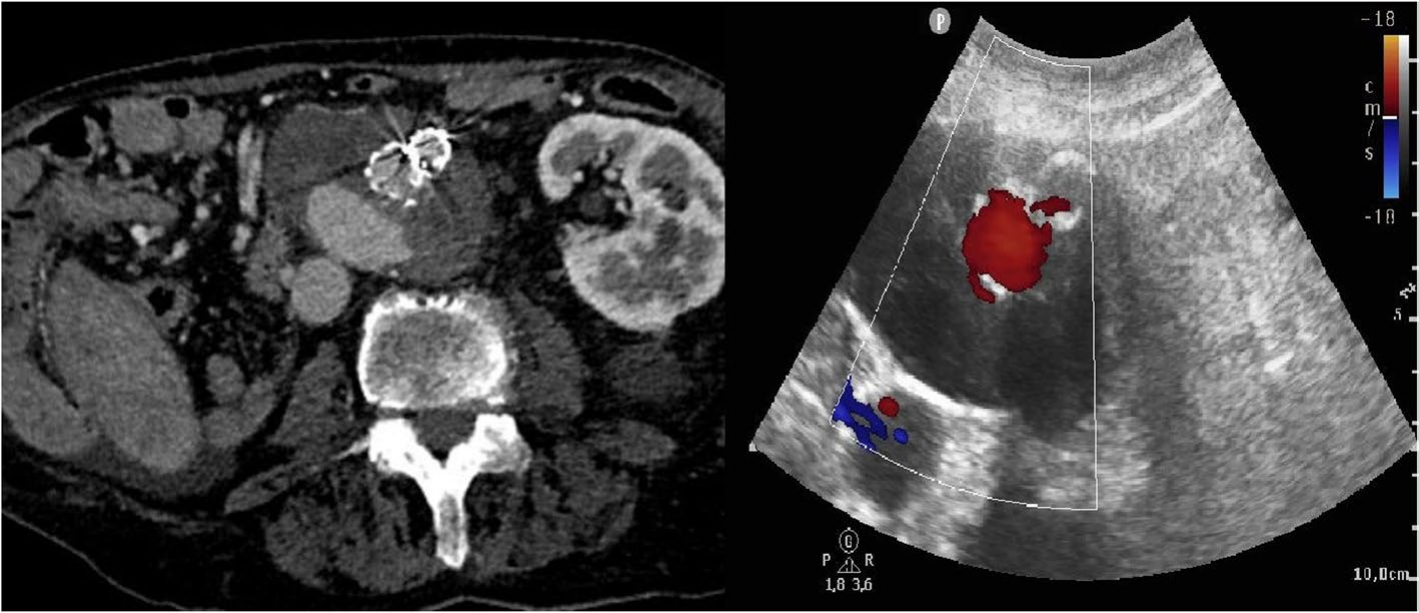
Type II endoleak demonstrated in the dorsal aspect of the aneurysm sac (CTA) (a). Transabdominal thrombin application was used to embolize type II endoleak (US) (b)

On CTA examination 4 days after the procedure, EL (30 × 30 mm in size) was detected in the dorsal aspect of the AAA sac. The washed-out residual at the lumbar artery origin was suggestive of a type II EL. Considering the EL extent, we opted for immediate treatment. Under local anesthesia with US guidance, the AAA sac puncture was performed through a transabdominal approach, and 1000 units of thrombin (Tisseel Lyo, Baxter, Germany) were injected sequentially (Fig. 3). A follow-up CT angiography (CTA) examination 2 months later showed complete thrombosis of the AAA sac with a slight regression in size and a patent renal SG. The patient remained free of AAA specific clinical complaints aside from an abdominal bulge and passed away 13 months after treatment due to cardiac failure.

## Discussion

In the ENGAGE registry, secondary EL was identified in 12% of patients after EVAR [7]. The formation of secondary ELs raises the need for long-term dispensary management of these patients [8]. Early invasive treatment is indicated when high-risk type I and III ELs are detected. According to the current recommendations of the European Society for Vascular Surgery, EV treatment is the preferred first choice [6]. In case of failure, surgical resection of AAA sac is considered, although it has higher morbidity and mortality rates, especially in polymorbid patients [9].

Various EV techniques can currently be used to treat type IA EL in aortic aneurysms, including proximal aortic SG extension, seal zone angioplasty, use of aortic stents, fenestrated or branched SGs, implantation of parallel SGs, and embolization with tissue glue or coils [8].

EV insertion of a tubular extension of the main SG body is increasingly being used for the urgent management in distal migration of the proximal SG end. In juxtarenal AAAs with insufficient anchorage zone below the RA origin, fenestrated proximal components can be used [10]. Fenestrated SGs are made to measure according to the dimensions obtained from CTA. The delivery time ranges from 6 to 8 weeks.

In the case of a symptomatic EL type IA with AAA sac size progression and impending rupture, urgent treatment with parallel SGs inserted into visceral arteries with simultaneous proximal extension of the main body of the SG (chimney technique) is possible [11]. This technique was chosen in our symptomatic patient because the equipment is readily available in the department and there is no danger of delay.

The disadvantage of the chimney technique compared to fenestrated and branched SGs is the more frequent occurrence of type IA EL between the parallel SGs. Therefore, it is not recommended to introduce more than two parallel SGs simultaneously and the distance between the AMS and AR should not be less than 2 cm [6]. These complications can be resolved by subsequent EV embolization or by direct percutaneous puncture of the AAA sac [11].

In order to ensure the SG tightness to treat EL IA and/or to prevent its occurrence, an aortic endostapler (Heli-FX, Medtronic Inc., USA) can be used to achieve internal–external fixation of the proximal section of the SG to the aortic wall using screws. According to available data, the method has a low complication rate [12], but it is not yet available in most specialized centres in the Czech Republic and often has anatomic limitations.

Type II EL is perceived as risky and is indicated for treatment only if it causes an enlargement of the AAA sac by more than 10 mm per year [6]. This type of EL can be resolved by transarterial embolization through communicating vessels using tissue glue and coils [8]. If the source is IMA, it can be retrogradely probed via collaterals from the SMA, but this approach is technically demanding and time consuming. In the case of early EL, the catheter can be introduced into the AAA sac by direct penetration along the wall of the inserted SG.

Another option is minimally invasive treatment by direct paravertebral puncture of the AAA sac under CT navigation or transabdominal puncture with the application of embolization materials [13]. This solution is a preferable therapeutic method, especially in polymorbid patients. In our patient, direct transabdominal puncture of the sac with thrombin application was chosen for the management of type II EL due to favorable anatomic conditions (the AAA sac was located near the abdominal wall without bowel loops) and based on our long term experience with percutaneous thrombin application [14].

If endovascular and minimally invasive methods fail, type II EL can be resolved by surgical methods, which include ligation or laparoscopic clipping on the source vessel or resection of the AAA sac [9].

## Conclusion

Successful treatment of symptomatic proximal type I EL with preservation of solitary AR perfusion was achieved in a polymorbid patient by implantation of an extension into the original, dislocated SG in combination with parallel insertion of the SG into the AR. The subsequent type II EL was successfully resolved under ultrasound guidance by direct percutaneous thrombin injection into the AAA sac. This chimney technique should be used in emergency situations where it is not possible to wait for fenestrated stent grafts. The transabdominal approach to endoleak embolization is a reasonable approach in an enlarged AAA sac with a close relationship to the abdominal wall. Our complication arose 7 years after primary successful EVAR therapy. The long-term stability of abdominal SGs after EVAR should be verified by regular follow-up with ultrasound and CTA to reliably detect EL types at risk of rupture and other potential complications of this treatment.

## Data Availability

Not applicable.

## Abbreviations

AAA: Abdominal aortic aneurym
EV: Endovascular
EVAR: Endovascular aoric repair
SG: Stent graft
EL: Endoleak
IMA: Inferior mesenteric artery
RA: Renal artery
SMA: Superior mesenteric artery
